# CAR-T versus allogeneic transplantation as consolidation for B-cell acute lymphoblastic leukemia in remission: a propensity-score matched study

**DOI:** 10.3389/fimmu.2026.1858385

**Published:** 2026-07-20

**Authors:** Fangfei Xu, Yulin Yang, Kuangguo Zhou, Xiuping Zou, Wei Huang

**Affiliations:** 1 Department of Hematology, Tongji Hospital, Tongji Medical College, Huazhong University of Science and Technology, Wuhan, China; 2 Suizhou Hospital, Hubei University of Medicine, Suizhou, China

**Keywords:** acute lymphoblastic leukemia, chimeric antigen receptor T-cell, complete remission, consolidation therapy, hematopoietic stem cell transplantation

## Abstract

**Background:**

Despite being a standard consolidation treatment, allogeneic hematopoietic stem cell transplantation (allo-HSCT) for B-cell acute lymphoblastic leukemia (B-ALL) in remission is still hampered by relapse and complications. While CAR-T therapy is revolutionizing later-line treatment, its role as a consolidation strategy in remission, particularly for patients ineligible for transplantation, remains unexplored. This study compared the efficacy of CAR-T therapy with that of allo-HSCT as consolidation for B-ALL patients in complete remission (CR).

**Methods:**

A retrospective, propensity-score matched study was performed from September 1, 2012 to December 31, 2024, including 75 B-ALL patients treated with CAR-T or allo-HSCT as consolidation therapy. Firth penalized likelihood Cox models and restricted mean survival time (RMST) models were built for time-to-event outcomes.

**Results:**

The 2-year progression-free survival (PFS) and 2-year overall survival (OS) rates showed no significant differences between the CAR-T and HSCT cohorts (CR1: 2-year PFS rate: 65.8% vs. 88.9%, P = 0.146, 2-year OS rate: 83.6% vs. 91.3%, P = 0.512; CR2: 2-year PFS rate: 42.2% vs. 64.3%, P = 0.360). The RMSTs of PFS and OS also have no significant differences (CR1: RMST of PFS difference = -7.738, P = 0.496, RMST of OS difference = -2.257, P = 0.788; CR2: RMST of PFS difference = -6.176, P = 0.700, RMST of OS difference = -8.391, P = 0.477) truncated at the minimum of the largest follow-up times in each cohort (CR1, 74.6 months; CR2, 75.0 months). In multivariate analysis, patients treated with allo-HSCT had better PFS in CR1 group (HR = 4.579, 95%CI, 1.020-20.563, P = 0.038). High risk stratification was an independent factor associated with worse OS for B-ALL patients in CR (CR1: HR = 8.010; 95%CI, 1.744-36.781, P = 0.048; CR2 HR = 5.110, 95%CI, 1.167-22.378, P = 0.004).

**Conclusion:**

The results indicated that CAR-T consolidation regimen showed comparable survival to that of allo-HSCT in matched B-ALL patients, which might support its potential as a consolidation alternative for B-ALL patients ineligible for allo-HSCT.

## Introduction

In the era of traditional chemotherapy, allogeneic hematopoietic stem cell transplantation (allo-HSCT) is an important treatment for consolidation of B-cell acute lymphoblastic leukemia (B-ALL) after achieving first complete remission (CR1) or second complete remission (CR2) (1, 2). However, relapse remains the leading cause of death in ALL patients after allo-HSCT. For B-ALL patients, the cumulative incidence of relapse (CIR) was 17.3%-30.2%, and the non-relapse mortality (NRM) ranged from 6.96% to 27.2% (3–8). Furthermore, many ALL patients could not receive allo-HSCT due to the lack of suitable donors and low remission rates achieved with traditional chemotherapy (9).

The emergence of new drugs, such as third-generation tyrosine kinase inhibitors (TKIs) and bispecific T-cell engagers (BiTEs), along with the application of pediatric non-transplant regimens, has begun to challenge the role of allo-HSCT as consolidation therapy for Philadelphia chromosome (Ph)- positive ALL patient (10–12).

In recent years, numerous studies have fully demonstrated that chimeric antigen receptor T cell (CAR-T) therapies were highly effective in relapsed or refractory ALL, with response rates ranging from 70% to 90% (13, 14). However, the high efficacy of CAR-T cell therapy did not always translate into long-term survival benefits, as indicated by the relapse rate of 38.7% and a median leukemia-free survival of only 6.1 months (15). High tumor burden in B-ALL was one of the principal factors contributing to relapse after CAR-T therapy (16). Reinfusion of CD19 CAR-T cells during remission after initial CD19 CAR-T therapy was a feasible treatment to reduce relapses and prolong disease-free survival in ALL (17). Anti-CD19 CAR-T consolidation therapy combined with TKI for Ph-positive ALL achieved good relapse-free survival (84%) and overall survival (83%) with a median follow-up of 27 months (18). We need to focus on the core issue of whether CAR-T could serve as an alternative consolidation regimen to allo-HSCT for patients with ALL in CR. AALL1721/Cassiopeia is the first single-arm trial for ALL patients in CR1 treated with CAR-T therapy (19). However, there are currently no cohort studies and few prospective studies to evaluate the differences in progression-free survival (PFS) and overall survival (OS) between CAR-T therapy and allo-HSCT for ALL patients in CR.

In this setting, we retrospectively investigated the clinical data of B-ALL patients in CR1 or CR2 who received CAR-T therapy or allo-HSCT from September 1, 2012 to December 31, 2024. In this cohort study, we compared the treatment outcomes of the B-ALL patients in CR1 or CR2 treated with CAR-T versus those treated with allo-HSCT. We aimed to determine whether CAR-T therapy could serve as a consolidation regimen in the allo-HSCT-free consolidation paradigms.

## Materials and methods

### Study population

The B-ALL patients treated with CAR-T or allo-HSCT after remission (including CR1 and CR2) were identified from the hospitalized case system of our center from September 1, 2012, to December 31, 2024. These patients were divided into a CR1 group and a CR2 group for separate analysis. Enrolled patients were assessed as being in complete remission based on bone marrow examination. CR was defined as the presence of less than 5% blasts in the bone marrow with the recovery of peripheral blood counts, and the disappearance of extramedullary lesions. Pediatric patients (< 14 years old) and patients whose treatment response could not be evaluated were excluded. Patients treated with both CAR-T and allo-HSCT were also excluded. The B-ALL patients were classified into standard-risk and high-risk ALL based on the patients’ clinical characteristics (age, white blood cell count, phenotype) and cytogenetics/molecular risk stratification at the time of initial diagnosis according to the NCCN guidelines (NCCN Guidelines Version 2.2025, Acute Lymphoblastic Leukemia). The flow cytometry with 8-color assay was performed to detect the measurable residual disease (MRD) in bone marrow specimens before CAR-T and allo-HSCT conditioning chemotherapy (20, 21). In the CR1 group, the period from CR to consolidation therapy was defined as the interval between the patient achieving first CR after diagnosis and receiving CAR-T or allo-HSCT; in the CR2 group, this period was defined as the interval from the first evidence of CR by bone marrow aspiration after relapse to the initiation of CAR-T or allo-HSCT.

### Treatments

The CAR-T therapies included CD19 CAR-T, CD22 CAR-T and CD19/C22 CAR-T therapies. Thirty-eight patients were enrolled either in prospective clinical studies or in compassionate-use programs because they did not conform to the inclusion criteria of these studies (NCT05388695, NCT05618041, NCT04916860, ChiCTR-OPN-16009847, ChiCTR-OPN-16008526, ChiCTR2000038641, ChiCTR1900023922). Before receiving CAR-T therapy or allo-HSCT, all patients had completed at least one traditional chemotherapy regimen recommended by the NCCN Guidelines, and Ph-positive patients were additionally treated with TKIs.

### Outcomes

Long-term follow-up was conducted, with the statistical cutoff date of September 1, 2025. The primary objectives of the study were PFS and OS after receiving CAR-T therapy or allo-HSCT. Disease relapse was defined as ≥5% blasts in the bone marrow and/or extramedullary relapse. PFS was defined as the time from CAR-T therapy or allo-HSCT to relapse, death, or the follow-up cutoff date. OS was defined as the time from CAR-T therapy or allo-HSCT to death or the follow-up cutoff date.

### Propensity score matching

All enrolled patients were classified into two cohorts: the CAR-T cohort and the HSCT cohort. To reduce the bias due to cohort selection, the propensity score matching (PSM) was used. Using logistic regression, PSM calculated a probability value, referred to as the propensity score (PS), corresponding to the independent variables. The following covariables were used to calculate the PS for matching: age group, gender, Ph status, number of prior chemotherapy regimens, risk stratification and MRD status before CAR-T or allo-HSCT. Matching was performed using the nearest neighbor method with a standard caliper width of 0.2 and with replacement, at a 1:3 ratio in the CR1 group and a 1:1 ratio in the CR2 group. This matching process successfully paired 55 patients in the CR1 group and 20 patients in the CR2 group, respectively. 103 patients in the two groups who were unmatched due to the caliper and matching ratio restrictions were excluded from subsequent statistical analysis. The standardized mean difference (SMD) was used to assess the balance of the matching. An absolute SMD of less than 0.2 was considered a negligible difference. After matching, most baseline characteristics had an absolute SMD of less than 0.2 (**Supplementary Table 1**). However, the absolute SMDs of risk stratification in the CR1 group and the Ph status in the CR2 group were 0.22 and 0.42, slightly exceeding 0.2 (**Supplementary Table 1**, **Supplementary Figure 1**). Nevertheless, these differences were considered acceptable, and the two groups were well matched after PSM.

### Statistical analysis

The differences in demographic and clinical variables between the two patient group were analyzed by independent samples t-test and Chi-square test, respectively. Kaplan-Meier curves were generated for PFS and OS with comparison by log-rank test. To address potential small-sample bias and the complete separation issues, Firth penalized likelihood Cox models were conducted to obtain more robust parameter estimates and confidence intervals. The restricted mean survival time (RMST) models were applied to address nonproportionality (e.g., survival curve crossover), truncated at 2 years and at the minimum of the largest follow-up times in each of the two cohorts (CR1 group, 74.6 months; CR2 group, 75.0 months) (22). All models were adjusted for the following covariates: gender, age, Ph status, number of prior chemotherapy regimens, risk stratification, MRD status before CAR-T or allo-HSCT, the period from CR to consolidation therapy and consolidation regimen (CAR-T or allo-HSCT). Statistical analyses were performed using R version 4.3.1. A P value < 0.05 was considered statistically significant.

## Results

### Patient baseline characteristics

**Figure 1:**
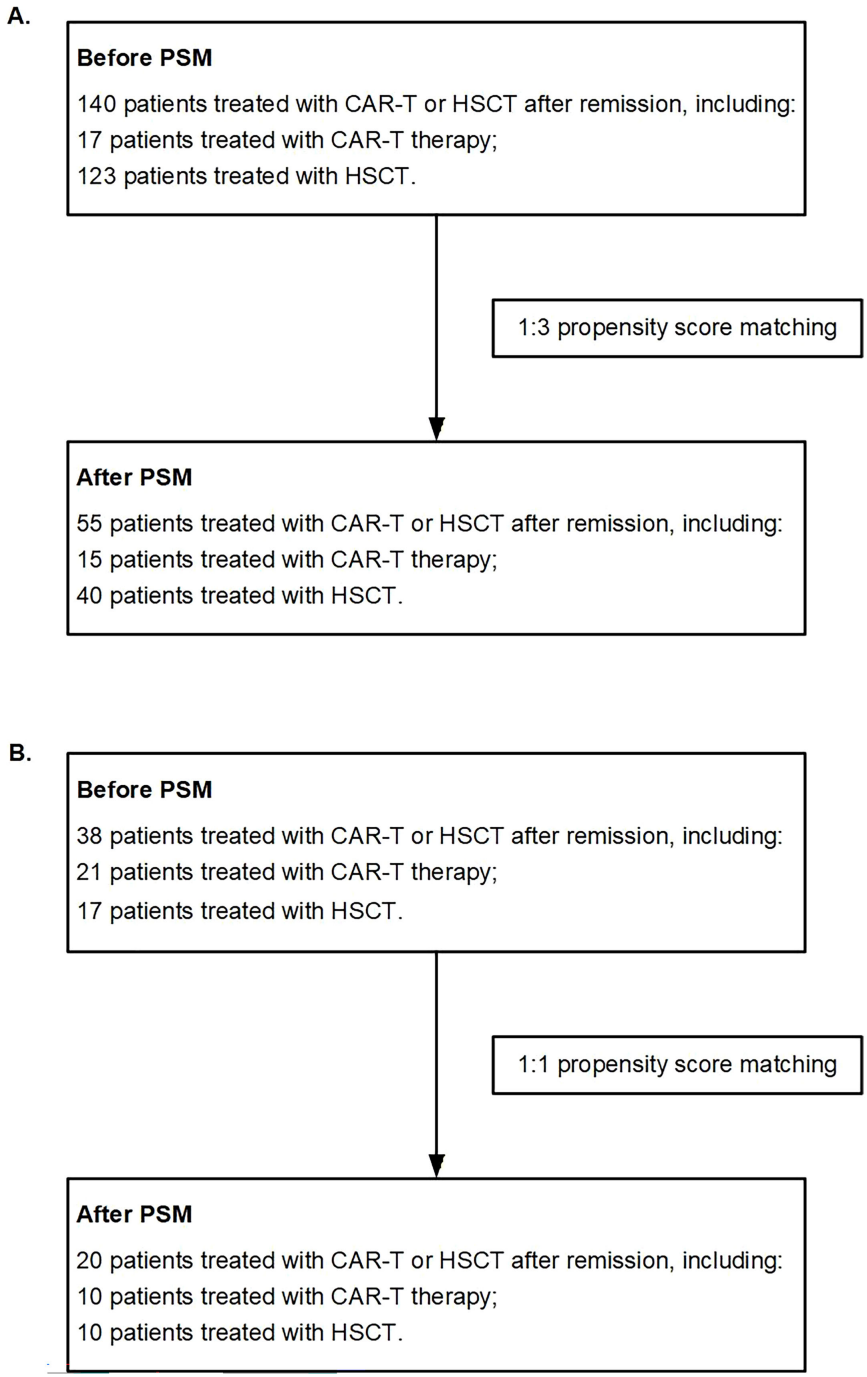
Flow algorithm for patient selection and analysis. **(A)** CR1 group; **(B)** CR2 group. CR, complete remission; PSM, propensity score matching; CAR-T, chimeric antigen receptor T-cell; HSCT, hematopoietic stem cell transplantation.

This study included 140 patients with CR1 and 38 patients with CR2. These patients were divided into the CAR-T cohort and the HSCT cohort in each remission group. Before PSM, the number of prior chemotherapy regimens these patients received in the CAR-T cohort were slightly more than that in the HSCT cohort in CR1 group (P = 0.003, **Supplementary Table 2**). After propensity score matching between these two cohorts, there were 55 patients and 20 patients in the CR1 group and the CR2 group, respectively (**Figure 1**). Patients’ characteristics were detailed in **Table 1**, and there were no differences in baseline characteristics between the two cohorts in each group. Among CR1 patients, 4 and 11 patients received CD19 CAR-T and CD19/CD22 CAR-T therapy respectively; among CR2 patients, 2 patients and 8 patients received CD19 CAR-T and CD19/CD22 CAR-T therapy, respectively. In the CR1 group, a total of 5 patients did not achieve MRD-negative CR before the treatment of CAR-T therapy or allo-HSCT (6.7% in the CAR-T cohort vs. 10.0% in the HSCT cohort; P = 1.000; **Table 1**). There were no significant differences in the percentage of patients with MRD-positive before treatment with CAR-T therapy or allo-HSCT among CR2 patients (20.0% in the CAR-T cohort vs. 20.0% in the HSCT cohort; P = 1.000; **Table 1**).

**Table 1:** Patient characteristics after propensity score matching.

Characteristics	CR1 group			CR2 group		
	CAR-T (n = 15)	HSCT (n = 40)	P	CAR-T (n = 10)	HSCT (n = 10)	P
Gender (M/F)	9/6	28/12	0.529	5/5	6/4	1.000
Age group, years, n, (%)			0.763			1.000
> 35	8(53.3%)	18(45.0%)		3(30.0%)	3(30.0%)	
≤ 35	7(46.7%)	22(55.0%)		7(70.0%)	7(70.0%)	
Ph-positive, n, (%)	7(46.7%)	23(57.5%)	0.551	1(10.0%)	3(30.0%)	0.582
Number of prior chemotherapy regimens, n, (%)			0.763			1.000
> 4	8(53.3%)	18(45.0%)		8(80.0%)	7(70.0%)	
≤ 4	7(46.7%)	22(55.0%)		2(20.0%)	3(30.0%)	
Risk stratification, n, (%)			0.558			1.000
Standard risk	9(60.0%)	20(50.0%)		8(80.0%)	7(70.0%)	
High risk	6(40.0%)	20(50.0%)		2(20.0%)	3(30.0%)	
MRD positive before CAR-T or HSCT, n, (%)	1(6.7%)	4(10.0%)	1.000	2(20.0%)	2(20.0%)	1.000
The period from CR to consolidation, median(range), months	16.80 (1.4-58.6)	4.70 (2.0-9.6)	0.001	2.00 (0.8-12.1)	2.55 (0.3-9.0)	0.684

Ph, Philadelphia chromosome; MRD, measurable residual disease; CAR-T, chimeric antigen receptor T-cell; HSCT, hematopoietic stem cell transplantation; CR, complete remission.

The period from CR to consolidation therapy in the CAR-T cohort was significantly longer than that in the HSCT cohort in the CR1 group (16.80 months vs. 4.70 months, P = 0.001; **Table 1**), but there was no significant difference in the CR2 group (2.00 months vs. 2.55 months, P = 0.684, **Table 1**). In the CR1 group, all Ph-negative patients received at least one course of conventional cytotoxic treatment after CR, including the VDCLP regimen (vindesine 2 mg on day 1, day 8, day 15, and day 22; daunorubicin 30–45 mg/m^2^ or idarubicin 6–10 mg/m^2^ on days 1–3; prednisone 60 mg/m^2^ on days 1–28; cyclophosphamide 600 mg/m^2^ on day 1 and day 15; pegylated asparaginase 2500 IU/m^2^ on day 1 and day 15), hyper-CVAD/MA regimen (cyclophosphamide 300 mg/m^2^, q12h on days 1–3; vincristine 2 mg on day 4 and day 11; doxorubicin 50 mg/m^2^ on day 4; dexamethasone 40 mg on days 1–4, and days 11–14; alternating with high-dose methotrexate 1 g/m^2^ on day 1 and cytarabine 3 g/m^2^, q12h on days 2–3), and CAM regimen (cyclophosphamide 0.8–1.0 g/m^2^ on day 1, cytarabine 75–100 mg/m^2^ on days 1–7, mercaptopurine 60–75 mg/m^2^ on days 1–7); most of Ph-positive patients received conventional cytotoxic treatment combined with TKIs, except for one patient in the CAR-T cohort, who only received imatinib in this period; two patients and seven patients received blinatumomab in the CAR-T cohort and the HSCT cohort, respectively. In the CR2 group, eight patients and three patients did not receive any chemotherapy after CR2 in the CAR-T cohort and the HSCT cohort, respectively; the other patients received at least one course of conventional cytotoxic treatment, and one of them also received blinatumomab.

### 2-year OS rate and 2-year PFS rate

Among CR1 patients, with a median follow-up time of 18.12 months (range 2.2-86.5 months), the 2-year PFS rates were 65.8% (95%CI, 0.421-1.000) and 88.9% (95%CI, 0.793-0.998) in the CAR-T cohort and the HSCT cohort, respectively (**Table 2**). Among CR2 patients, no significant difference in the 2-year PFS rate between the two cohorts was observed (CAR-T vs. HSCT: 42.2% vs. 64.3%, P = 0.360; **Table 2**), with a median follow-up time of 25.10 months (range 2.9-148.9 months). Since no deaths occurred among patients whose follow-up periods exceeded two years, the 2-year OS was not calculated in CR2 group.

**Table 2:** Multivariate analysis of 2-year PFS rate and OS rate.

Covariate	CR1 group				CR2 group	
	2-year PFS rate (95%CI)	P	2-year OS rate (95%CI)	P	2-year PFS rate (95%CI)	P
Gender		0.913		0.617		0.283
Male	83.5%(0.699, 0.970)		90.9%(0.811, 1.009)		65.6%(0.335, 0.977)	
Female	82,2%(0.639, 1.005)		85.6%(0.669, 1.042)		40.0%(0.059, 0.741)	
Age group (years)		0.086		0.142		0.626
> 35	92.9%(0.794, 1.063)		96.2%(0.888, 1.035)		44.4%(0.009, 0.880)	
≤ 35	74.1%(0.575, 0.907)		84.0%(0.696, 0.984)		57.5%(0.286, 0.864)	
Ph status		0.819		0.087		0.518
Ph- positive	83.9%(0.689, 0.989)		96.6%(0.899, 1.032)		37.5%(0.043, 0.737)	
Ph- negative	81.3%(0.647, 0.979)		79.9%(0.620, 0.978)		57.9%(0.316, 0.843)	
Number of prior chemotherapy regimens		0.262		0.104		0.184
> 4	76.4%(0.580, 0.948)		81.7%(0.652, 0.981)		61.8%(0.348, 0.888)	
≤ 4	88.9%(0.771, 1.008)		96.4%(0.896, 1.033)		26.7%(0.022, 0.633)	
Risk stratification		0.314		0.593		0.601
Standard risk	88.6%(0.764, 1.007)		91.7%(0.806, 1.029)		57.0%(0.306, 0.835)	
High risk	77.8%(0.601, 0.954)		86.9%(0.729, 1.007)		40.0%(0.051, 0.754)	
MRD status before CAR-T or HSCT		0.850		0.382		0.311
MRD positive	80.0%(0.449, 1.151)		66.7%(0.133, 1.200)		75.0%(0.326, 1.174)	
MRD negative	83.6%(0.722, 0.949)		90.8%(0.820, 0.995)		49.0%(0.218, 0.761)	
Cohort		0.146		0.512		0.360
CAR-T	65.8%(0.421, 1.000)		83.6%(0.625, 1.047)		42.2%(0.082, 0.762)	
HSCT	88.9%(0.793, 0.998)		91.3%(0.818, 1.008)		64.3%(0.314, 0.972)	

Ph, Philadelphia chromosome; MRD, measurable residual disease; CAR-T, chimeric antigen receptor T-cell; HSCT, hematopoietic stem cell transplantation; CR, complete remission; PFS, progression-free survival; OS, overall survival.

### OS and PFS

**Figure 2:**
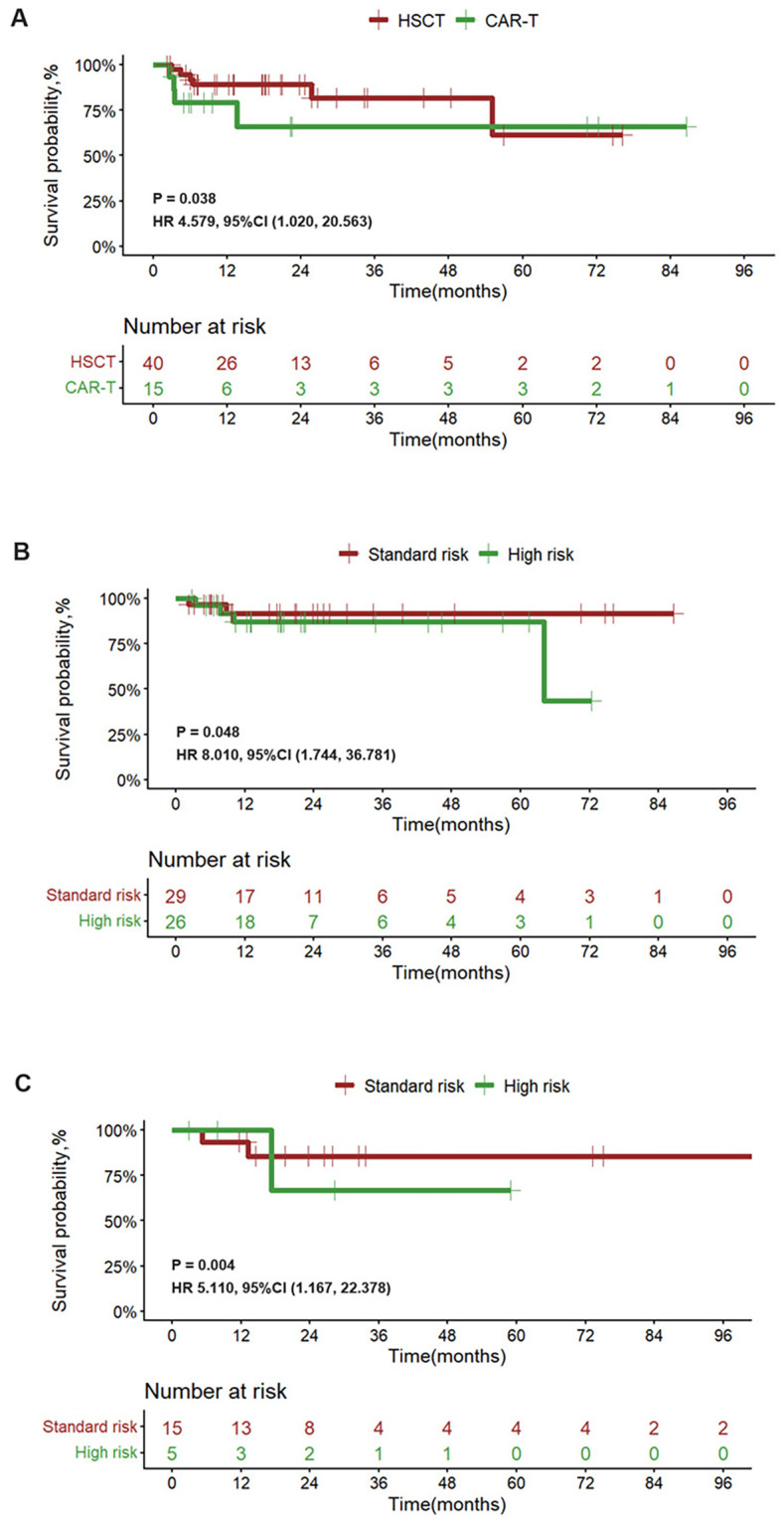
Kaplan-Meier curves for PFS and OS. **(A)** PFS for CAR-T cohort and HSCT cohort in the CR1 group; **(B)** OS for risk stratification in the CR1 group; **(C)** OS for risk stratification in the CR2 group. PFS, progression-free survival; OS, overall survival; CR, complete remission; CAR-T, chimeric antigen receptor T-cell; HSCT, hematopoietic stem cell transplantation; HR, hazard ratio.

**Supplementary Table 3** showed the results of the univariate Cox regression analysis with Firth penalized likelihood of the relationships between clinical factors and overall outcomes, and there were no significant differences in PFS or OS. However, in the multivariate analysis, patients treated with allo-HSCT showed increased PFS in the CR1 group (HR = 4.579; 95%CI, 1.020-20.563; P = 0.038; **Table 3**, **Figure 2A**). High risk stratification was associated with worse OS in both the CR1 group (HR = 8.010, 95%CI, 1.744-36.781; P = 0.048; **Table 3**, **Figure 2B**) and CR2 group (HR = 5.110, 95%CI, 1.167-22.378; P = 0.004; **Table 3**, **Figure 2C**). However, in the CR2 group, no significant differences in PFS and OS were observed between the CAR-T cohort and HSCT cohort (PFS: HR = 4.511, 95%CI, 0.620-32.891, P = 0.137; OS: HR = 3.014, 95%CI, 0.105-86.202; P = 0.519; **Table 3**).

**Table 3:** Multivariate Cox regression analysis with Firth penalized likelihood of PFS and OS.

Covariate	CR1 group				CR2 group			
	PFS		OS		PFS		OS	
	HR(95%CI)	P	HR(95%CI)	P	HR(95%CI)	P	HR(95%CI)	P
Gender	0.525 (0.107, 2.575)	0.427	0.399 (0.041, 3.897)	0.430	1.869 (0.221, 15.653)	0.568	0.699 (0.040, 12.289)	0.807
Age group	0.161 (0.026, 1.019)	0.052	0.978 (0.092, 10.444)	0.985	7.003 (0.605, 81.058)	0.119	0.874 (0.006, 137.165)	0.958
Ph status	2.574 (0.230, 28.769)	0.443	0.135 (0.003, 5.655)	0.294	1.117 (0.039, 32.065)	0.948	2.107 (0.017, 263.521)	0.838
Number of prior chemotherapy regimens	3.764 (0.507, 27.967)	0.195	1.484 (0.100, 21.968)	0.774	0.762 (0.021, 1.487)	0.075	0.487 (0.002, 157.888)	0.807
Risk stratification	2.741 (0.431, 17.398)	0.285	8.010 (1.744, 36.781)	0.048*	0.310 (0.010, 9.172)	0.498	5.110 (1.167, 22.378)	0.004*
MRD status before CAR-T or HSCT	8.326 (0.893, 77.660)	0.063	2.051 (0.148, 28.441)	0.592	0.131 (0.005, 3.437)	0.125	0.523 (0.003, 92.853)	0.865
Period from CR to consolidation	1.016 (0.937, 1.100)	0.705	1.038 (0.931, 1.156)	0.504	0.736 (0.425, 1.275)	0.275	0.981 (0.569, 1.691)	0.946
Cohort	4.579 (1.020, 20.563)	0.038*	2.401 (0.326, 17.669)	0.390	4.511 (0.620, 32.819)	0.137	3.014 (0.105, 86.202)	0.519

Ph, Philadelphia chromosome; MRD, measurable residual disease; CAR-T, chimeric antigen receptor T-cell; HSCT, hematopoietic stem cell transplantation; CR, complete remission; PFS, progression-free survival; OS, overall survival; HR, hazard ratio. * P < 0.05

At 2 years, the RMSTs of PFS and OS in the HSCT cohort were both slightly higher than those in the CAR-T cohort, but the differences were not significance (PFS: P = 0.162 in the CR1 group, P = 0.208 in the CR2 group; OS: P = 0.464 in the CR1 group, P = 0.263 in the CR2 group; **Table 4**). Similarly, at 74.6 months in the CR1 group and 75.0 months in the CR2 group, the RMSTs of PFS and OS also showed no significant differences (PFS: P = 0.496 in the CR1 group, P = 0.700 in the CR2 group; OS: P = 0.788 in the CR1 group, P = 0.477 in the CR2 group; **Table 4**).

**Table 4:** The RMST test for PFS and OS.

	CR1 group			CR2 group		
	CAR-T	HSCT	P	CAR-T	HSCT	P
RMST at 2 years (months)						
PFS	18.246(13.513, 22.980)	21.900(19.954, 23.845)		15.218(9.584, 20.853)	19.831(15.370, 24.293)	
OS	21.015(17.211, 24.819)	22.554(20.979, 24.130)		20.540(16.210, 24.870)	23.163(21.627, 24.698)	
PFS difference	-3.653(-8.771, 1.464)		0.162	-4.613(-11.800, 2.574)		0.208
OS difference	-1.539(-5.656, 2.578)		0.464	-2.623(-7.217, 1.971)		0.263
RMST at largest follow-up time (months)						
PFS	51.547(32.182, 70.913)	59.286(48.222, 70.349)		36.734(14.369, 59.099)	42.910(20.873, 64.947)	
OS	63.302(57.667, 73.451)	65.559(57.667, 73.451)		59.397(40.436, 78.357)	67.787(54.564, 81.011)	
PFS difference	-7.738(-30.041, 14.564)		0.496	-6.176(-37.574, 25.222)		0.700
OS difference	-2.257(-18.732, 14.219)		0.788	-8.391(-31.507, 14.725)		0.477

RMST, restricted mean survival time; PFS, progression-free survival; OS, overall survival; CAR-T, chimeric antigen receptor T-cell; HSCT, hematopoietic stem cell transplantation.

## Discussion

Over recent decades, tremendous progress has been made in improving the survival of B-ALL patients. Allo-HSCT has been recommended as a crucial consolidation therapy for patients who achieve a response to chemotherapy, but the high recurrence rate after allo-HSCT remains unsatisfactory (23). In recent years, with the intensification of the conditioning regimen, 2-year LFS and OS rates were 45.3%-94.44% and 50.6%-89.72% for ALL patients after allo-HSCT, respectively (3–9, 24–41). In our study, the 2-year PFS and OS rates in the CR1 group were 88.9% and 91.3%, respectively, which are consistent with previous studies. Beyond traditional chemotherapy, cellular immunotherapy offers new hope for further reducing MRD in ALL, potentially allowing patients to avoid allo-HSCT (42). Studies about the combination of TKIs with BiTE, or TKIs with CAR-T therapy, represent promising attempts at consolidation therapy for Ph-positive ALL without the need for allo-HSCT (10, 18).

In our study, patients in CAR-T cohort and HSCT cohort were matched based on age (> 35 years, ≤ 35 years), gender, Ph status (Ph-positive, Ph-negative), number of prior chemotherapy regimens (> 4 lines, ≤ 4 lines), risk stratification and MRD status before CAR-T or allo-HSCT, and two groups were well matched (**Supplementary Table 1**, **Supplementary Figure 1**). The period from CR to consolidation therapy in the CAR-T cohort was significantly longer than that in the HSCT cohort for the CR1 group (16.80 months vs. 4.70 months). In contrast, for the CR2 group, the period was slightly shorter in the CAR-T cohort than that in the HSCT cohort (2.00 months vs. 2.55 months). This may be attributed to the fact that patients in the CR1 group tended to receive more chemotherapy prior to CAR-T consolidation therapy, with the aim of consolidating remission status and minimizing MRD levels. Patients in the CR2 group were at high risk of relapse again, so effective therapy strategies were urgently needed to consolidate their remission status. The 2-year OS rate in the CAR-T cohort in the CR1 group was 83.6%, similar to that of the HSCT cohort and comparable to the OS rate reported for B-ALL patients after allo-HSCT in previous studies (4, 8). Moreover, there were no significant differences in the RMSTs of PFS and OS between the two cohorts. Overall, CAR-T may serve as an alternative consolidation regimen to allo-HSCT with similar survival outcomes. Patients in the HSCT cohort showed increased PFS in the CR1 group; however, the difference in 2-year PFS rate between the two cohorts was not significant in the CR1 group. This may be due to the relatively short follow-up time of the study. Therefore, long-term follow-up studies are still needed to assess recurrence in B-ALL patients treated with CAR-T as a consolidation regimen.

High risk factors, including high cytogenetic risk, MRD positive before consolidation treatment, could increase the recurrence rate (6, 28, 43, 44). Multivariate analysis in our study suggested that high cytogenetics/molecular risk was an independent prognostic factor for poor survival (CR1 group: HR = 8.010, 95%CI, 1.744-36.781, P = 0.048; CR2 group: HR = 5.110, 95%CI, 1.167-22.378, P = 0.004). This aligns with previous studies indicating that personalized and optimized therapy based on risk stratification could enhance overall survival (45).

Our research also has several limitations. As a retrospective study rather than a randomized prospective controlled trial, the treatment choices for CAR-T and allo-HSCT were not randomize and were influenced by various factors, including patient and physician preferences as well as economic and social considerations. Although the PSM was conducted, selection bias could not be ignored, as there were unmatched variables that were difficult to evaluate between the two cohorts, such as the patients’ physical condition and organ function. Achieving case balance may result in substantial sample loss, thereby affecting the generalizability and statistical power of the results. In this study, differences in CAR-T products and inclusion criteria across clinical studies may affect the outcomes of CAR-T treatment; however, subgroup analysis of CAR-T products could not be performed due to the small sample size. This was a single-center study, which also limits the generalizability of the results.

In conclusion, this study suggested that CAR-T consolidation regimen showed comparable survival to that of allo-HSCT in matched B-ALL patients, supporting the feasibility of the CAR-T consolidation regimen as part of allo-HSCT-free consolidation strategies for B-ALL. The CAR-T consolidation regimen may become a promising treatment option for B-ALL patients in CR, especially those at high risk of GVHD. However, larger prospective studies with longer follow-up periods are warranted to confirm these findings. Additionally, questions remain regarding the recurrence after CAR-T consolidation therapy in B-ALL patients.

## Data Availability

The original contributions presented in the study are included in the article/**Supplementary Material**. Further inquiries can be directed to the corresponding authors.
