# Supplementary material for: CAR-T versus allogeneic transplantation as consolidation for B-cell acute lymphoblastic leukemia in remission: a propensity-score matched study
Source: Front Immunol. 2026 Jul 20;17:1858385. doi: 10.3389/fimmu.2026.1858385 (PMC13429391; doi:10.3389/fimmu.2026.1858385)

**Supplementary Material**

**Contents**

**Supplementary Table 1. The standardized mean difference before and after propensity score matching.**

**Supplementary Table 2. Patient characteristics before propensity score matching.**

**Supplementary Table 3. Univariate Cox regression analysis with Firth penalized likelihood of PFS and OS.**

**Supplementary Figure 1. The love plot of the standardized mean difference.**

**Supplementary Table 1. The standardized mean difference before and after propensity score matching.**

| Characteristics | CR1 Group | | CR2 Group | |
| --- | --- | --- | --- | --- |
|  | Unmatched | Matched | Unmatched | Matched |
| Gender | 0.0602 | 0.2032 | 0.1066 | 0.2002 |
| Age group | 0.4849 | 0.0903 | -0.1846 | 0.0000 |
| Ph status | 0.1610 | -0.2003 | -0.0416 | -0.4243 |
| Number of prior chemotherapy regimens | 0.7327 | 0.0452 | 0.2790 | 0.2214 |
| Risk stratification | 0.3401 | -0.2226 | -0.0416 | -0.2121 |
| MRD positive before CAR-T or HSCT | -0.3028 | -0.0944 | 0.3720 | 0.0000 |

Ph: Philadelphia chromosome; MRD: measurable residual disease; CAR-T: Chimeric antigen receptor T-cell; HSCT: hematopoietic stem cell transplantation; CR: complete remission.

**Supplementary Table 2. Patient characteristics before propensity score matching.**

| Characteristics | CR1 Group | | | CR2 Group | | |
| --- | --- | --- | --- | --- | --- | --- |
|  | CAR-T  (n = 17) | HSCT  (n = 123) | P | CAR-T  (n = 21) | HSCT  (n = 17) | P |
| Gender (M/F) | 10/7 | 76/47 | 1.000 | 10/11 | 9/8 | 1.000 |
| Age group, years, n, (%) |  |  | 0.067 |  |  | 0.743 |
| > 35 | 10(58.8%) | 43(35.0%) |  | 8(38.1%) | 8(47.1%) |  |
| ≤ 35 | 7(41.2%) | 80(65.0%) |  | 13(61.9%) | 9(52.9%) |  |
| Ph-positive, n, (%) | 8(47.1%) | 48(39.0%) | 0.601 | 7(33.3%) | 6(35.3%) | 1.000 |
| Number of prior chemotherapy regimens, n, (%) |  |  | **0.003** |  |  | 0.502 |
| > 4 | 10(58.8%) | 28(22.8%) |  | 15(71.4%) | 10(58.8%) |  |
| ≤ 4 | 7(41.2%) | 95(77.2%) |  | 6(28.6%) | 7(41.2%) |  |
| Risk stratification, n, (%) |  |  | 0.174 |  |  | 1.000 |
| Standard risk | 9(52.9%) | 86(69.9%) |  | 14(66.7%) | 11(64.7%) |  |
| High risk | 8(47.1%) | 37(30.1%) |  | 7(33.3%) | 6(35.3%) |  |
| MRD positive before CAR-T or HSCT, n, (%) | 1(5.9%) | 16(13.0%) | 0.694 | 6(28.6%) | 2(11.8%) | 0.257 |

Ph: Philadelphia chromosome; MRD: measurable residual disease; CAR-T: Chimeric antigen receptor T-cell; HSCT: hematopoietic stem cell transplantation; CR: complete remission.

**Supplementary Table 3. Univariate Cox regression analysis with Firth penalized likelihood of PFS and OS.**

| Variable | CR1 Group | | | | CR2 Group | | | |
| --- | --- | --- | --- | --- | --- | --- | --- | --- |
|  | PFS | | OS | | PFS | | OS | |
|  | HR(95%CI) | P | HR(95%CI) | P | HR(95%CI) | P | HR(95%CI) | P |
| Gender | 0.848  (0.220, 3.261) | 0.810 | 0.980  (0.182, 5.286) | 0.982 | 1.716  (0.459, 6.422) | 0.422 | 0.684  (0.066, 7.082) | 0.750 |
| Age group | 0.391  (0.098, 1.561) | 0.187 | 0.495  (0.084, 2.910) | 0.437 | 1.984  (0.528, 7.454) | 0.310 | 0.305  (0.010, 9.341) | 0.496 |
| Ph status | 1.065  (0.302, 3.764) | 0.922 | 0.444  (0.083, 2.363) | 0.341 | 3.261  (0.820, 12.958) | 0.093 | 2.468  (0.236, 25.830) | 0.451 |
| Number of prior chemotherapy regimens | 1.292  (0.373, 4.483) | 0.686 | 1.981  (0.373, 10.534) | 0.423 | 0.311  (0.073, 1.313) | 0.112 | 0.589  (0.056, 6.180) | 0.659 |
| Risk stratification | 2.344  (0.617, 8.899) | 0.211 | 2.038  (0.378, 10.996) | 0.408 | 1.656  (0.360, 7.611) | 0.517 | 2.208  (0.213, 22.901) | 0.507 |
| MRD status before CAR-T or HSCT | 3.179  (0.699, 14.454) | 0.135 | 3.250  (0.429, 24.643) | 0.254 | 0.697  (0.108, 4.482) | 0.703 | 0.460  (0.015, 14.191) | 0.657 |
| Period from CR to consolidation | 1.014  (0.953, 1.079) | 0.206 | 1.036  (0.988, 1.086) | 0.145 | 0.809  (0.614, 1.067) | 0.134 | 0.860  (0.541, 1.368) | 0.526 |
| Cohort | 2.054  (0.573, 7.361) | 0.269 | 1.458  (0.261, 8.137) | 0.668 | 1.528  (0.407, 5.732) | 0.530 | 2.121  (0.203, 22.143) | 0.530 |

Ph: Philadelphia chromosome; MRD: measurable residual disease; CAR-T: Chimeric antigen receptor T-cell; HSCT: hematopoietic stem cell transplantation; PFS: progression-free survival; OS: overall survival; HR: hazard ratio.

**Supplementary Figure 1. The love plot of the standardized mean difference. (A). CR1 group; (B) CR2 group.** CR: complete remission; MRD: minimal residual disease; CAR-T: chimeric antigen receptor T cell; HSCT: hematopoietic stem cell transplantation; Ph: Philadelphia chromosome.


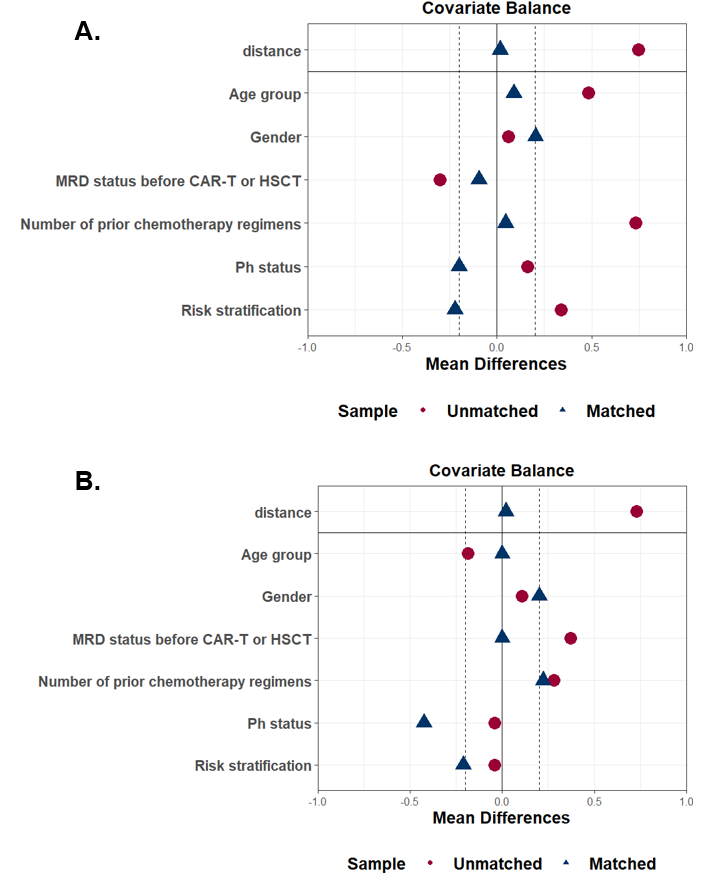

Supplement: Supplementary file 1 [file DataSheet1.docx]
